# Supervised Treatment Interruptions Fail to Control HIV-1 Viremia

**DOI:** 10.1371/journal.pmed.0010048

**Published:** 2004-10-26

Highly active antiretroviral therapy (HAART) for the treatment of individuals infected by HIV-1 is limited by high costs, drug resistance, and drug-related toxicities. This has led researchers to investigate new treatment options, including ways to boost immune responses to better control HIV. One such approach has been termed supervised treatment interruption (STI)—in which HAART is intermittently stopped once viral load has been reduced to a low level, in order to boost natural immunity by brief exposure to virus. The goal is to allow for the eventual discontinuation of drug treatment.[Fig pmed-0010048-g001]

**Figure pmed-0010048-g001:**
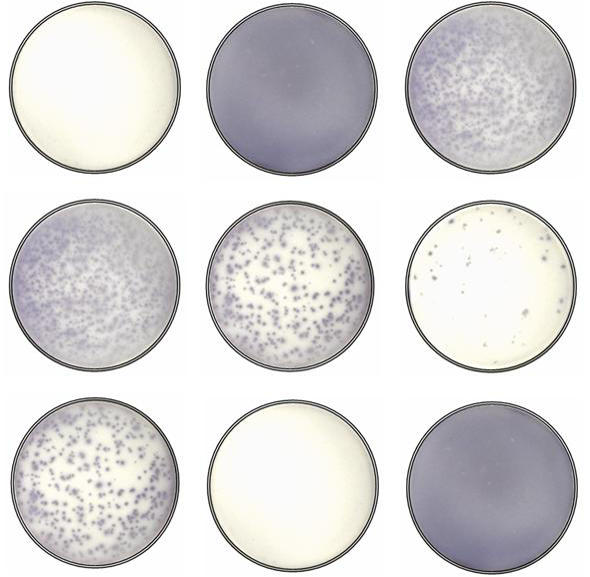
ELISPOT assays detect HIV-specific cytotoxic T lymphocyte responses

Preliminary evidence, published by Bruce Walker and his colleagues from Harvard Medical School in *Nature* in 2000, suggested that this approach worked in persons treated in the earliest stages of acute HIV infection. HIV-1 viral loads in newly infected patients remained suppressed for a median of six months after therapy had been stopped. However, a follow up paper, published this month in *PLoS Medicine* by the same research group, shows that the viral load rebounded in eight of the 14 patients by one year.

“The findings are very straightforward and very important,” comments Danny Douek from the Vaccine Research Center, National Institutes of Health, United States, who was not involved in the study. “In almost every case, virus rebounded and no clinical benefit from the interruption could be determined.”

Walker's team first considered the possibility of STI in 1997 after they demonstrated that HAART given to patients recently infected with HIV could protect T helper cells, which are normally destroyed in the earliest stages of infection. They hypothesized that early treatment of acute HIV-1 infection with HAART might boost the immune response, allowing it to control the HIV-1 infection without the need for continuous therapy. “We did not know at that time whether the T helper cells would be functional,” explains Walker. “The only way to tell this was to stop medications and see if the immune response could control the virus.”

To test this hypothesis the researchers did an open-label trial of STIs; they published data from six months follow-up in the *Nature* paper. “The key finding was that we were able to get at least transient control of virus in all eight persons studied, and in five of eight the viral load was less than 500 copies (very low!) at the time of publication,” explains Walker. However, at that point they did not know how long the protective effects would last.

The first evidence that protection was not complete came two years later when Walker's team reported a case of superinfection; one of the patients in the original experiment was infected with a second strain of HIV, even though the first virus was still well controlled. “This paper was important because it indicated that the amount of immunity might be enough for the person's own virus, but might not protect against closely related viruses circulating in the population,” says Walker.

The *PLoS Medicine* study adds more concern since it shows that although most persons can indeed transiently control their own virus, they do so for only a limited amount of time. “We expanded the study to 14 persons, and now have about five years of follow-up on some of the patients,” says Walker. “Although we were able to use early treatment and structured treatment interruption to boost immunity and have 11 of 14 patients control their virus, most of the persons ultimately ‘broke through,’ meaning that they had a recurrence of viremia.” At the present time the researchers do not know what causes the loss of viral control.

Walker and colleagues conclude that treatment interruptions should probably be avoided outside the setting of controlled clinical trials, whereas Douek goes a step further: “The study shows that even early short-term treatment and structured treatment interruptions, using current strategies, impart only transient benefit and are unlikely to serve as a reasonable therapeutic option in the future.”

